# Stellungnahme der Österreichischen Gesellschaft für Pneumologie (ÖGP): Management von Patienten mit SARS-CoV-2-Infektionen und von Patienten mit chronischen Lungenerkrankungen während der COVID-19-Pandemie (Stand 27.04.2020)

**DOI:** 10.1007/s00740-020-00350-4

**Published:** 2020-05-18

**Authors:** H. Flick, B. M. Arns, J. Bolitschek, B. Bucher, K. Cima, E. Gingrich, S. Handzhiev, M. Hochmair, F. Horak, M. Idzko, P. Jaksch, G. Kovacs, R. Kropfmüller, B. Lamprecht, J. Löffler-Ragg, M. Meilinger, H. Olschewski, A. Pfleger, B. Puchner, C. Puelacher, C. Prior, P. Rodriguez, H. Salzer, P. Schenk, O. Schindler, I. Stelzmüller, V. Strenger, H. Täubl, M. Urban, M. Wagner, F. Wimberger, A. Zacharasiewicz, R. H. Zwick, E. Eber

**Affiliations:** 1 Klinische Abteilung für Pulmonologie, Univ. Klinik für Innere Medizin, Medizinische Universität Graz/LKH Graz Ost, Graz, Österreich; 2 1. Medizinische Abteilung, Hanusch Krankenhaus, Wien, Österreich; 3 Ordensklinikum Elisabethinen Linz, Linz, Österreich; 4 Abteilung für Pneumologie, Tirol Kliniken, Landeskrankenhaus Hochzirl-Natters, Natters, Österreich; 5 Lungenfachärztliche Ordination, Wien, Österreich; 6 Klinische Abteilung für Pneumologie, Universitätsklinikum Krems, Krems, Österreich; 7 Karl Landsteiner Institut für Lungenforschung und pneumologische Onkologie, Krankenhaus Nord – Klinik Floridsdorf, Wien, Österreich; 8 Allergiezentrum Wien West, Wien, Österreich; 9 Klinische Abteilung für Pulmologie, Univ. Klinik für Innere Medizin II, Medizinische Universität Wien/AKH Wien, Wien, Österreich; 10 Klinische Abteilung für Thoraxchirurgie, Univ. Klinik für Chirurgie, Medizinische Universität Wien/AKH Wien, Wien, Österreich; 11 Ludwig Boltzmann Institut für Lungengefäßforschung Graz, Graz, Österreich; 12 Klinik für Lungenheilkunde/Pneumologie, Medizinische Fakultät, Johannes Kepler Universität, Linz, Österreich; 13 Pneumologische Ambulanz, Univ. Klinik für Innere Medizin II, Medizinische Universität Innsbruck, Innsbruck, Österreich; 14 Abteilung für Innere Medizin und Pneumologie, Krankenhaus Nord – Klinik Floridsdorf, Wien, Österreich; 15 Klinische Abteilung für pädiatrische Pulmonologie und Allergologie, Univ. Klinik für Kinder- und Jugendheilkunde, Medizinische Universität Graz, Auenbruggerplatz 34/2, 8036 Graz, Österreich; 16 Department für Pneumologie, Reha Zentrum Münster, Münster, Österreich; 17 Interdisziplinäres Schlaflabor, Telfs, Österreich; 18 Lungenfachärztliche Ordination, Innsbruck, Österreich; 19 Abteilung Pulmologie, Landesklinikum Hochegg, Grimmenstein, Österreich; 20 Abteilung für Innere Medizin und Pneumologie, LKH Graz II, Standort Enzenbach, Gratwein, Österreich; 21 Lungenfachärztliche Ordination, Salzburg, Österreich; 22 Abteilung für Kinder- und Jugendheilkunde, Wilhelminenspital der Stadt Wien, Lehrkrankenhaus der Medizinischen Universität Wien, Wien, Österreich; 23 Ambulante Pneumologische Rehabilitation, Therme Wien Med, Wien, Österreich

**Keywords:** SARS-CoV‑2, COVID-19, Ambulant erworbene Pneumonie, „Acute Respiratory Distress Syndrome“ (ARDS), Chronische Lungenerkrankungen, SARS-CoV‑2, COVID-19, Community-acquired pneumonia, ARDS, Chronic lung diseases

## Abstract

Die COVID-19-Pandemie stellt derzeit weltweit eine Herausforderung dar. In Österreich konnte eine Krise innerhalb des medizinischen Versorgungssystems bisher verhindert werden. Die Behandlung von Patienten mit ambulant erworbener Pneumonie („community acquired pneumonia“, CAP), inklusive durch SARS-CoV-2-Infektionen, sollte sich auch während der Pandemie weiterhin an evidenzbasierten CAP-Leitlinien orientieren. COVID-19-spezifische Anpassungen sind jedoch sinnvoll. Die Behandlung von Patienten mit chronischen Lungenerkrankungen muss während der Pandemie angepasst werden, ist aber weiterhin zu gewährleisten.

## Abstract (en)

The COVID-19 pandemic is currently a challenge worldwide. In Austria, a crisis within the health care system has so far been avoided. The treatment of patients with community-acquired pneumonia (CAP), including SARS-CoV‑2 infections, should continue to be based on evidence-based CAP guidelines during the pandemic. However, COVID-19-specific adjustments are useful. The treatment of patients with chronic lung diseases must be adapted during the pandemic, but must still be guaranteed.

## Einleitung

Mit der COVID-19-Pandemie steht das österreichische Gesundheitssystem aktuell unverändert vor einer großen Herausforderung. Auf allen Ebenen wurden seit März 2020 in hohem Tempo einschneidende Anpassungen in den bisher gewohnten medizinischen Versorgungsstrukturen und -abläufen durchgeführt, um auf eine große Zahl von akut schwer an COVID-19 erkrankten Patienten vorbereitet zu sein. Gleichzeitig konnte mit einschneidenden gesamtgesellschaftlichen Präventivmaßnahmen die Geschwindigkeit der SARS-CoV-2-Ausbreitung in Österreich abgebremst und eine kritische Überlastung der medizinischen Versorgungszentren zunächst erfolgreich verhindert werden.

In der aktuellen Situation bestehen für die Pneumologie drei Ziele:

1. Optimale medizinische Behandlung von schwer an COVID-19 erkrankten Patienten, um eine möglichst niedrige SARS-CoV-2-Letalitätsrate zu erreichen.
2. Gewährleistung einer unverändert bestmöglichen medizinischen Akutversorgung von Patienten mit anderen schweren pulmonalen Krankheitsbildern (Infektionen, Asthma‑, COPD-, ILD- oder CF-Exazerbationen, Lungenembolie, malignitätsverdächtige pulmonale Raumforderungen u. a.).
3. Fortsetzung wichtiger medizinischer Behandlungen von Menschen mit vorbestehenden schweren chronischen Grunderkrankungen (Lungenkarzinom, Asthma, COPD, pulmonale Hypertension, ILD, CF, St. p. Lungentransplantation, schlafbezogene Atemstörungen u. a.). Diese Patienten bedürfen unserer besonderen Aufmerksamkeit, da sie durch eine SARS-CoV‑2 Infektion zusätzlich bedroht sein können.

Zum Erreichen aller drei genannten Ziele sollten wir uns im ärztlichen Handeln unverändert, soweit mit den aktuell pandemiebedingt limitierten Ressourcen möglich, an vorhandenen evidenzbasierten und gut implementierten Leitlinien orientieren und diese im Einzelfall an die aktuell schwierige Situation anpassen. Besonders bei chronischen Erkrankungen bedarf dies Augenmaß und einer offenen Kommunikation mit Patienten und Angehörigen, um praktikable Lösungen zu finden.

## Management von Patienten mit SARS-CoV-2-Infektionen

### Aktuelle epidemiologische Situation

#### Allgemeines zu COVID-19

Die COVID-19-Pandemie hat sich seit Jänner 2020 rasch global ausgebreitet. Weltweit sind laut WHO 2.804.796 COVID-19-Fälle bestätigt und 193.710 Patienten bereits daran verstorben [1].

Epidemiologische Informationen und Studienergebnisse zu COVID-19 müssen zurzeit noch zurückhaltend interpretiert werden. Sie unterliegen einer starken Dynamik und multifaktoriellen Einflüssen, weisen eine variable Datenqualität auf und sind im internationalen Vergleich aufgrund versorgungsstruktureller und epidemiologischer Unterschiede nur eingeschränkt vergleichbar. Wie beispielsweise beim „Antibiotic Stewardship“ üblich, sollten nationale und regionale Daten systematisch erfasst und regelmäßig ausgewertet werden. Nur so kann die aktuelle lokale Situation ausreichend beurteilt werden.

Es handelt sich bei der SARS-CoV-2-Infektion, ähnlich wie bei der Influenza, um eine Virusinfektion mit variablem Verlauf (von asymptomatisch über mild und schwer bis letal). Bei den meisten in Europa positiv getesteten Personen liegt eine milde Symptomatik vor. Hospitalisierte Personen klagen hingegen in ≥80 % über Fieber, Husten und Atemnot (Tab. 1; [2, 3]). Hierbei handelt es sich meist um ältere und komorbide Patienten. In vielen Fällen liegt eine durch SARS-CoV‑2 ambulant erworbene Pneumonie („community acquired pneumonia“, CAP) mit daraus resultierender Hypoxie vor. Zusätzlich werden COVID-19-spezifische Phänomene beschrieben, wie ein herabgesetztes Empfinden von Dyspnoe, wodurch womöglich eine respiratorische Verschlechterung subjektiv lange nicht wahrgenommen wird, eine fehlende Steigerung der Atemfrequenz trotz schwerer Oxygenierungsstörung und ein passagerer Geruchs- und Geschmacksverlust.

**Table 1:** 

Symptome	Positiv getestete Personen (inklusive milde Fälle; [%])	Hospitalisierte COVID-19 Patienten [%]
Fieber	49	85
Husten	24	86
Atemnot	–	80
Muskelschmerzen	–	34
Durchfall	2	27
Übelkeit/Erbrechen	–	24
Halsschmerzen	12	18
Kopfschmerzen	–	16
Schnupfen	4	16
Brustschmerzen	–	15
Bauchschmerzen	–	8
Schwäche, allgemein	8	–
Schmerzen, allgemein	7	–

Aufgrund der Infektiosität des Erregers ist perspektivisch auch mit krankenhausassoziierten SARS-CoV-2-Pneumonien zu rechnen.

Entsprechend dem „European Centre for Disease Prevention and Control“ (ECDC) wurden bis jetzt in Europa schwere COVID-19-Verläufe (Notwendigkeit der Hospitalisierung) im Median bei 28 % aller Fälle beobachtet. Durch unentdeckte milde Verläufe ist jedoch von einer hohen Dunkelziffer und einer höheren Rate milder Verläufe auszugehen.

Bei hospitalisierten Patienten lag im Median bei 16 % der Fälle eine sehr schwere Erkrankung (Notwendigkeit einer intensivmedizinischen Behandlung oder respiratorischen Unterstützung) vor und die COVID-19-Krankenhausletalität liegt in Europa derzeit bei 14 % [2].

Bezüglich der COVID-19-Todesfälle pro 100.000 Einwohner gibt es in Europa relevante Differenzen. So wurden bei vergleichbarer COVID-19-Inzidenz (155–170 Fälle/100.000 Einwohner) in Österreich und Deutschland bis jetzt 5 Todesfälle/100.000 und in Frankreich, Großbritannien bzw. den Niederlanden 19–27 Todesfälle/100.000 registriert [4]. Dazu passend wurde durch das EuroMOMO-Netzwerk (European monitoring of excess mortality for public health action) in bestimmten europäischen Ländern (Großbritannien, Schweden, Frankreich, Spanien, Belgien, den Niederlanden, Italien und der Schweiz) eine sehr starke pandemieassoziierte Übersterblichkeit erfasst, deutlich weniger jedoch in Österreich und anderen Ländern wie Deutschland, Norwegen, Irland und Dänemark [5].

In Österreich wurden bisher 15.239 Personen SARS-CoV-2-positiv getestet und 549 (3,6 %) sind an oder mit COVID-19 verstorben. Aktuell (Stand 27.04.2020) sind 579 COVID-19-Patienten hospitalisiert (Höchststand Anfang März mit 1010 hospitalisierten Patienten), davon werden 140 auf Intensivstationen behandelt (Höchststand Anfang März mit 267 ICU-Patienten; 24–26 % – also deutlich mehr als im europäischen Durchschnitt). Damit waren in Österreich Anfang März 26 % (aktuell 12 %) aller verfügbaren Intensivbetten mit COVID-19-Patienten belegt [6]. Primärdaten zur Zahl der bisher im Krankenhaus oder auf Intensivstationen behandelten Patienten und deren Letalitätsrate liegen aus Österreich derzeit nicht vor.

#### Hospitalisierungs- und Sterblichkeitsrisiko bei COVID-19 und anderen erregerbedingten ambulant erworbenen Pneumonien

Um die aktuellen COVID-19-Daten realistisch einordnen zu können, müssen diese auch mit der Häufigkeit und dem Verlauf schwerer respiratorischer Infektionen vor der Pandemie verglichen werden. Prinzipiell gilt, dass erregerbedingte CAP häufig sind. Bei einer Inzidenz von 296 krankenhauspflichtigen CAP pro 100.000 Einwohnern werden in Österreich jährlich schätzungsweise 26.222 und monatlich 2185 Patienten mit einer CAP im Krankenhaus behandelt [7]. Bei einer durchschnittlichen Krankenhaus-Letalitätsrate von 13 % (Tab. 2) ergeben sich für Österreich jährlich 3409 (39 CAP-Todesfälle/100.000) und monatlich 284 CAP-Todesfälle. An COVID-19 verstarben während der Hochphase der Pandemie (27.03. bis 27.04.2020) 491 Menschen pro Monat. Daher ist anzunehmen, dass es im Rahmen der Pandemie mindestens zu einer passageren Verdoppelung der CAP Todesfälle/100.000 Einwohner kam.

**Table 2:** 

	Krankenhausletalität (%)	ICU-Letalität (%)
*CAP allgemein* [7, 15–19]	12,9–14,1	17,0–29,5
*S. pneumoniae* [18, 20, 21]	8,0–12,0	17,5–26,0
*L. pneumonia* [22–25]	3,9–18,5	21,6
*Virale CAP allgemein* [26, 27]	14,8	22,0
*Influenza A/B* [10, 19, 28–31]	12,6	17,1–41,2
*COVID-19*		
China (Wuhan)^a^ [32–36]	10,7–21,9	61,5
USA (New York)^b^ [37]	21,0	78,0
Europa (ECDC) [2]	14	–
Großbritannien^a^ [26]	–	30,3–50,7
Spanien^a^ [38]	–	29,2
Italien (Lombardei)^a^ [39]	–	25,6

^a^Epizentren der COVID-19-Pandemie

^b^Epizentrum New York: am 23.04.2020 ca. 10-mal mehr SARS-CoV-2-Infizierte/100.000 Einwohner und 20-mal mehr COVID-Todesopfer/100.000 Einwohner als zum selben Zeitpunkt in Österreich [40]

Die Influenza muss getrennt betrachtet werden, da die Influenza-Letalität nicht ausschließlich durch Influenza-Pneumonien bedingt ist, aber jährlich weltweit mit 400.000 Influenza-assoziierten Todesfällen gerechnet wird [8, 9].

Die Inzidenz krankenhauspflichtiger Influenza-Erkrankungen liegt in Europa je nach Saison und effektiver Durchimpfungsrate der Bevölkerung bei 12–95/100.000 und allein bei Kindern in Österreich von 2002–2018 bei 50/100.000 [10–13]. Wird diese Inzidenz auf Österreich im Allgemeinen übertragen und dabei von einer ICU-Rate von 7 % und einer Krankenhaus-Letalität von 4 % ausgegangen, ergeben sich während jeder Influenza-Saison in Österreich mindestens 1152 bis 8416 krankenhauspflichtige Erkrankungen, 81 bis 589 ICU-pflichtige Erkrankungen und 46 bis 337 im Krankenaus verstorbene Patienten. Für die Zeit von Dezember bis April (Influenza-Saison) bedeutet dies für Österreich monatlich 288 bis 2104 krankenhauspflichtige und 20 bis 147 ICU-pflichtige Influenza-Erkrankungen. Aufgrund der im Vergleich mit anderen europäischen Ländern sehr niedrigen Influenza-Durchimpfungsrate ist in Österreich eher mit einer höheren als niedrigeren Rate zu rechnen. Diese Annahme wird durch Berechnungen der AGES gestützt, die basierend auf dem FluMOMO-Modell in den letzten 4 Jahren von durchschnittlich 2326 Influenza-Todesopfern pro Jahr und somit während der Influenza-Saison von 582 Influenza-Todesopfern pro Monat ausgeht (COVID-19: aktuell ca. 450 Tote pro Monat, Stand 19.04.2020; [14]). Somit führt in Österreich die jährliche Influenza-Welle sehr wahrscheinlich zu einer vergleichbaren Belastung des Gesundheitswesens wie die derzeitige COVID-19-Pandemie. Eine systematische Erfassung von Influenza-assoziierten Todesfällen hospitalisierter Patienten sollte daher, wie aktuell für COVID-19 etabliert, auch in Österreich eingeführt werden.

Zusammenfassend kann davon ausgegangen werden, dass mit SARS-CoV‑2 ein weiterer relevanter CAP-Erreger hinzugekommen ist, der für eine noch nicht absehbare Zeit die CAP-Inzidenz besonders bei älteren Menschen deutlich erhöht und wie bei Influenza-Infektionen mit einem erheblichen krankenhaushygienischen und logistischen Aufwand verbunden ist. Aufgrund der staatlich verordneten Präventivmaßnahmen in und außerhalb des Gesundheitswesens wurde die COVID-19-Pandemie in Österreich zwar vorerst erfolgreich eingedämmt, eine erneute Zunahme an COVID-19-Fällen ist nach Lockerung der Maßnahmen jedoch möglich. Soweit aktuell beurteilbar, scheint die Hospitalisierungsrate bei SARS-CoV-2-CAP höher als bei und die Krankenhaus-Letalität in Abhängigkeit von der Funktionalität des Gesundheitssystems vergleichbar mit anderen erregerbedingten CAP zu sein (Tab. 2).

Das Letalitätsrisiko einer CAP wird bestimmt vom Ausmaß der unmittelbaren Lungenparenchymschädigung, sekundären Infektionen/Komplikationen, dem Alter und vorbestehenden Komorbiditäten sowie der Qualität der verfügbaren medizinischen Versorgung. Die Bedeutung klassischer kardiopulmonaler, renaler und metabolischer Komorbiditäten für den Verlauf einer CAP sind von Influenza‑, Pneumokokken- und Legionella-Infektionen bekannt und spielen in gleicher Weise auch bei der SARS-CoV-2-CAP eine entscheidende Rolle. So steigt das Hospitalisierungs- und Sterblichkeitsrisiko bei der SARS-CoV-2-CAP ähnlich wie bei anderen CAP-Erregern ab dem 60. Lebensjahr und mit der Zahl der Begleiterkrankungen deutlich an (Tab. 3; [7, 10, 15, 29, 41]).

**Table 3:** 

Komorbiditäten verstorbener Patienten	COVID-19 (%)	Andere CAP-Erreger (%)
Arterieller Hypertonus	40–75	54
Diabetes	20–31	31
Herzerkrankungen	23–49	38
Neurologische Erkrankungen	13	16–19
Karzinome	2–18	28
Chronische Niereninsuffizienz	23	13–27
Chronische Lungenerkrankungen	8–19	6–24
Demenz	18	28

Bei COVID-19 wurde darüber hinaus deutlich, dass die Letalitätsrate einer akuten Erkrankung immer auch von gesellschaftlichen und strukturellen Faktoren bestimmt wird (beispielsweise zeitgerechte Public-Health-Interventionen zur Verlangsamung der Ausbreitungsgeschwindigkeit einer pandemischen Infektion, zeitnahe und flexible Strukturanpassung des Gesundheitssystems, Zahl der akut verfügbaren Intensiv- oder Beatmungsbetten, Kapazität an Isolations- und Schutzmöglichkeiten im ambulanten und stationären Bereich, kurzfristige und effektive Schulung des medizinischen Personals). In einigen Ländern und Regionen kam es zu akuten Versorgungsnotständen. Es ist anzunehmen, dass in diesen krisenhaften und teilweise katastrophenmedizinischen Situationen nicht alle akut schwer erkrankten Patienten rechtzeitig und adäquat medizinisch versorgt werden konnten. So lag beispielsweise die Letalitätsrate im primär unvorbereiteten Epizentrum (Stadt Wuhan in der Provinz Hubei) zunächst bei 12 % und in den anderen chinesischen Provinzen später nur noch bei ca. 1 % [45]. Auch die von EuroMOMO erfasste und oben genannte Übersterblichkeit in einigen von der Pandemie stark betroffenen Ländern deutet darauf hin.

#### SARS-CoV-2 bei Kindern

In einer Auswertung der ersten knapp 45.000 Labor-bestätigten COVID-19-Fälle in China stellten Kinder <10 Jahren nur 0,9 % (416 Kinder) und Kinder zwischen 10 und 19 Jahren nur 1,2 % (549 Kinder) der Fälle dar [46]. Neonatale COVID-19-Erkrankungen wurden bisher extrem selten beobachtet [47]. Wie viele Kinder tatsächlich infiziert sind, aber aufgrund fehlender oder milder Symptomatik nicht getestet werden, ist unbekannt. Enger Kontakt mit einem SARS-CoV-2-Erkrankten im familiären Umfeld scheint der häufigste Übertragungsweg zu sein [48].

Im Vergleich zu Erwachsenen erkranken Kinder und Jugendliche wesentlich seltener an SARS-CoV‑2 und zeigen häufig nur milde klinische Symptome. Nur ein Viertel entwickelt Temperaturen zwischen 38 und 39 °C, nur 10 % Temperaturen >39,0 °C. Husten und Tachypnoe werden in ca. 30–50 % beschrieben und Pharyngitis (5–45 %), Rhinitis (10–30 %), Diarrhoe (10–30 %) sowie Erbrechen (6 %) deutlich seltener [48–51]. Laborchemisch fallen ähnlich wie bei Erwachsenen eine Erhöhung von CRP (mäßig), Transaminasen, LDH, D‑Dimer und CK sowie eine Leukopenie (in erster Linie Lymphopenie) auf [51].

Aufgrund der bei Kindern weniger spezifischen Symptome ist es schwierig, eine sichere klinische Diagnose zu stellen. Daher ist es gerade bei pädiatrischen Patienten wichtig, großzügig auf SARS-CoV‑2 zu testen und entsprechende Schutzmaßnahmen für das betreuende Personal umzusetzen.

Schwere Verläufe mit respiratorischer Insuffizienz oder der Notwendigkeit einer intensivmedizinischen Behandlung sind eher die Ausnahme [47]. Bei Säuglingen wurden wiederholt schwere COVID-19-Erkrankungen suspiziert, wobei es sich dabei meist um Verdachtsfälle (ohne SARS-CoV-2-Nachweis) handelte. Die Autoren gehen davon aus, dass ein nicht unbeträchtlicher Teil dieser schweren Verläufe durch andere Viren (v. a. RSV) verursacht gewesen sein könnte [52]. In der Literatur wurden bisher nur wenige pädiatrische COVID-19-Todesfälle berichtet [46, 47, 53].

Aufgrund der oft milderen Verläufe bei Kindern wurde diskutiert, ob oligo- und asymptomatische Kinder eine wesentliche Rolle in der Transmission spielen könnten, ohne dass diese Hypothese jemals wissenschaftlich bestätigt wurde [52]. Eine rezente Studie aus Island zeigt im Gegenteil, dass in einem Screening bei symptomlosen Personen der Anteil der Virusausscheider bei den 40- bis 50-Jährigen dreimal so hoch ist (ca. 1,5 %) wie bei Kindern/Jugendlichen zwischen 10 und 20 Jahren (ca. 0,5 %). Von über 800 getesteten Kindern unter 10 Jahren wurde kein einziges Kind positiv getestet [54].

SARS-CoV-2-Infektionen bei Kindern mit Risikofaktoren und Grunderkrankungen (chronische respiratorische Erkrankungen wie zystische Fibrose, schweres Asthma, bronchopulmonale Dysplasie sowie kardiale Erkrankungen, primäre und sekundäre Immundefizienz, maligne Grunderkrankung, Malnutrition etc.) werden in den bisherigen pädiatrischen Analysen kaum berichtet [46, 52]. Fraglich ist, ob sich daraus ableiten lässt, dass diese Kinder weniger gefährdet sind als Erwachsene mit Risikofaktoren, oder ob Kinder aus Risikogruppen effizienter vor Ansteckung geschützt werden konnten.

#### Epidemiologischer Ausblick

Sobald die staatlich verordneten Maßnahmen zur Pandemie-Eindämmung liberalisiert werden, muss das Gesundheitswesen nicht nur auf eine wieder steigende Zahl von COVID-19-Fällen vorbereitet sein. Auch alle anderen übertragbaren respiratorischen Infektionen (beispielsweise Influenza‑, RSV-, Pneumokokken-, *Mycoplasma*- und *Bordetella-*Infektionen), deren Ausbreitung durch die staatlichen Pandemie-Maßnahmen in gleicher Weise wie SARS-CoV‑2 passager unterdrückt wurde, werden wieder vermehrt auftreten.

**Figure 1:**
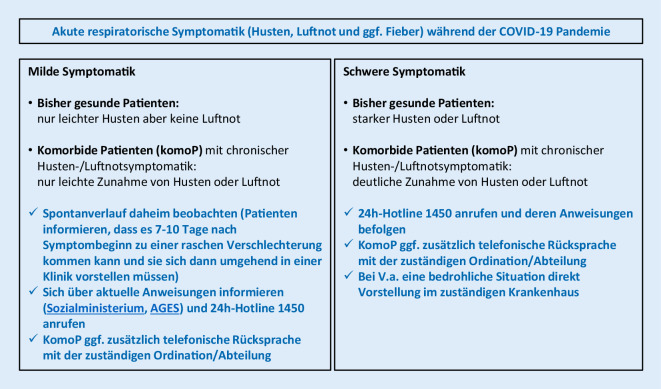

In diesem Zusammenhang ist das in der Bevölkerung durch die COVID-19-Pandemie gewachsene Bewusstsein zu potenziell bedrohlichen Infektionskrankheiten zu begrüßen. Vernünftige individuelle und gesellschaftliche Präventivmaßnahmen sollten jetzt zielgerichtet weiterentwickelt und gefördert werden. Dazu zählt beispielsweise die individuelle Bereitschaft zur Schutzimpfung gegen Influenza und andere relevante Erreger, aber auch ein vertieftes Wissen in der Bevölkerung zur selbstständigen Differenzierung zwischen harmlosen Infektionen, die zu Hause auskuriert werden sollten, und ernsthaften akuten Erkrankungen, die vom Hausarzt oder im Krankenhaus behandelt werden müssen (Abb. 1).

### Management der SARS-CoV-2-Pneumonie

#### Basis-Management der SARS-CoV-2-CAP

Bei der schweren SARS-CoV-2-Pneumonie handelt es sich um eine schwere, viral bedingte ambulant erworbene Pneumonie (svCAP), die vom klinischen Bild (akuter Beginn, bilaterale Pneumonie, progredientes respiratorisches Versagen, hohes Letalitätsrisiko) mit einer schweren Influenza-CAP vergleichbar ist (Tab. 2). Von entscheidender Bedeutung in der aktuellen Pandemie-Situation ist die Gewährleistung einer suffizienten medizinischen Versorgung derartig schwerer Krankheitsbilder. Aufgrund der Häufigkeit von svCAP (vor allem während der jährlichen Influenza-Saison) sind die medizinischen Zentren in Österreich mit dem klinischen Management von svCAP vertraut.

Da die Funktionalität des österreichischen Gesundheitssystems zur Versorgung akuter Erkrankungen während der bisherigen COVID-19-Pandemie nicht wesentlich beeinträchtigt wurde, sollten auch bei der SARS-CoV-2-CAP die bisher gültigen und evidenzbasierten Leitlinien zur Behandlung der CAP in ihren Kernpunkten zur Anwendung kommen und als Orientierung dienen [55, 56] (Abb. 1, 2 und 3):

- Frühzeitige Diagnose einer CAP, von möglicherweise gleichzeitig dekompensierten Grunderkrankungen und Erkennung von lebensbedrohlichen Situationen (Stichpunkt CAP als Notfall)
- Beginn der CAP-Therapie ohne zeitliche Verzögerung (inkludiert Therapie einer respiratorischen Insuffizienz, einer hämodynamischen Instabilität, von dekompensierten Grunderkrankungen und, wenn indiziert, eine antiinfektive Therapie)
- Triage entsprechend den klinischen Befunden (ambulante vs. stationäre vs. intensivmedizinische Therapie)
- Formulierung angemessener Therapieziele und Vermeidung aussichtsloser Übertherapien bei bereits schwer vorerkrankten Palliativpatienten (s. unten)
- In allen Phasen von Beginn an Einhaltung der strikten Hygienemaßnahmen zum Personalschutz und der Vermeidung nosokomialer Infektionen bei ansteckenden Erregern
- Prävention erneuter Infektionen

**Figure 2:**
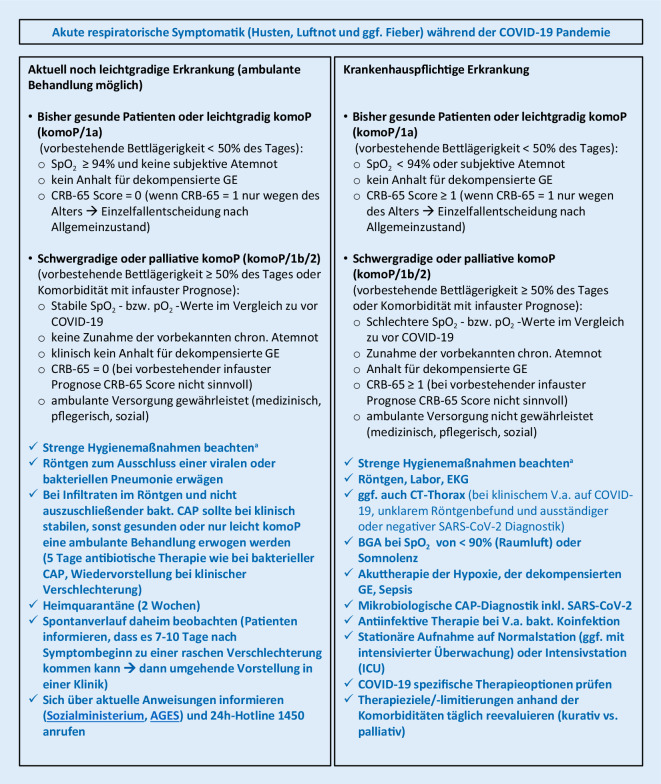

**Figure 3:**
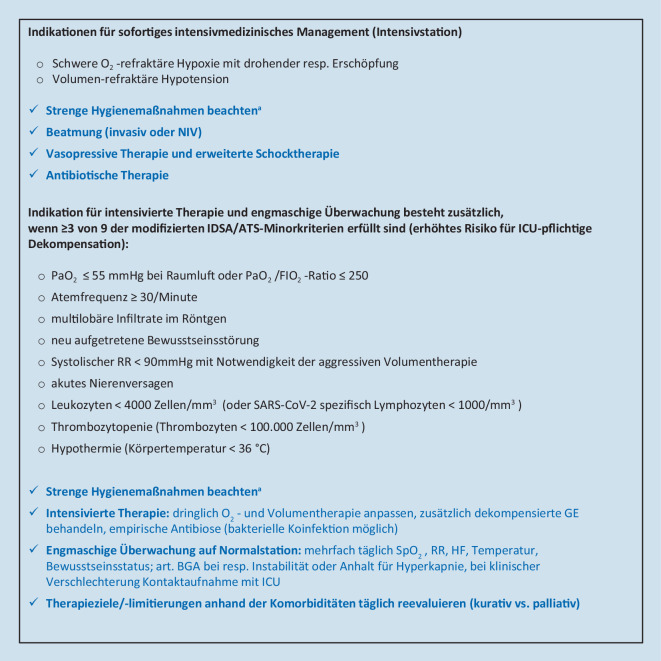

#### Diagnostik

Die Kernsymptome fast aller Atemwegsinfektionen sind Husten, ggf. Fieber und Dyspnoe. Dies gilt für jede Form einer bakteriellen oder viralen akuten Bronchitis, COPD-Exazerbation und Pneumonie inklusive COVID-19. Auch wenn aktuell in den Krankenhäusern vermehrt COVID-19-Erkrankungen diagnostiziert werden, sind andere akute kardiorespiratorische Erkrankungen und Infektionen ebenso präsent und werden nach Liberalisierung der gesamtgesellschaftlichen Pandemie-Maßnahmen ebenfalls wieder zunehmen. Daher ist eine ausschließlich COVID-19-zentrierte CAP-Diagnostik nicht sinnvoll. Vielmehr sollte sich die ambulante CAP-Diagnostik unverändert an den bisherigen Empfehlungen (z. B. der trinationalen S3-Leitlinie für Erwachsene aus dem Jahr 2016 und der trinationalen S2-Leitlinie für Kinder und Jugendliche aus dem Jahr 2017) orientieren [55, 56].

Trotzdem sind im Rahmen der COVID-19-Pandemie diagnostische Ergänzungen zur frühzeitigen Erkennung einer SARS-CoV-2-Infektion in der Routinediagnostik notwendig (Abb. 1, 2 und 3):

- Im ambulanten Bereich sollten während der Pandemie nicht schwer erkrankte Patienten mit den eingerichteten zuständigen Stellen telefonisch Kontakt aufnehmen und sich über das aktuelle Vorgehen informieren und beraten lassen (Abb. 1).
- Bei Patienten mit dem klinischen Bild einer möglichen Infektion der Atemwege ohne klare ätiologische Zuordnung sollte in Notaufnahmen oder im Krankenhaus bei therapeutischer oder medizinhygienischer Relevanz eine SARS-CoV-2-PCR durchgeführt werden (Abb. 2 und 3).
- Bei Patienten in der Notaufnahme oder bereits hospitalisierten Patienten mit dem klinischen Bild einer möglichen fieberhaften Infektion der tiefen Atemwege
  *und*
  – unauffälligem (oder schwer interpretierbarem) Röntgen-Bild
  *und*
  – negativer Schnelltestdiagnostik (Influenza‑/RSV-/SARS-CoV‑2 PCR, Pneumokokken-/*Legionella*-Schnelltest)
  *und*
  – COVID-19-typischen laborchemischen Veränderungen (Leukozyten <10,0 × 10^9^/l, Neutrophile <7,0 × 10^9^/l, Lymphozyten <1,0 × 10^9^/l, CRP nur mäßig erhöht [10–130 mg/l], Procalcitonin <1,0 ng/ml [34, 37])
  sollte die Indikation zu einer CT des Thorax ohne Kontrastmittel großzügig gestellt werden (Kontrastmittel nur notwendig, wenn zusätzlich eine Lungenembolie ausgeschlossen werden soll).

Bei einem COVID-19-typischen CT-Befund, aber negativer SARS-CoV-2-PCR sollte der Patient zunächst als COVID-19-Verdacht medizinhygienisch eingestuft, andere Differenzialdiagnosen proaktiv evaluiert und die SARS-CoV-2-PCR zeitnah wiederholt werden.

Eine positive SARS-CoV-2-PCR sichert die Diagnose COVID-19. Die Sensitivität einer virusspezifischen PCR ist dagegen multifaktoriell vom Testzeitpunkt (Beginn der Infektion vs. späterer Zeitpunkt), dem Probenmaterial (Rachen- vs. Nasopharynx-Abstrich vs. Sputum oder Bronchiallavage), der Probenqualität und dem verwendeten Testverfahren (unterschiedliche Assays) abhängig. Ein negativer PCR-Befund schließt daher COVID-19 bei sonst passender Klinik und typischem CT-Befund nicht aus. Sputumproben oder Bronchiallavage-Flüssigkeiten sind prinzipiell sensitiver für SARS-CoV‑2 als Nasopharynx-Abstriche [57]. Aus medizinhygienischen Bedenken sollten aber einzig zur COVID-19-Diagnosestellung weder eine Sputuminduktion noch eine diagnostische Bronchoskopie durchgeführt werden.

Beim intubierten Patienten empfiehlt sich bei klinischem Verdacht und initial negativem Abstrich der oberen Atemwege eine weitere Erregerdiagnostik mittels PCR aus den tieferen Atemwegen (z. B. Trachealsekret via geschlossenem Absaugsystem). Hierdurch wird die diagnostische Sensitivität erhöht und die falsch-negative Testrate reduziert [58, 59].

Das Thorax-Röntgen ist für die Diagnosestellung einer SARS-CoV-2-CAP weder ausreichend sensitiv noch spezifisch. Bei passender klinischer Symptomatik und positivem PCR-Ergebnis ist ein COVID-19-typischer Röntgenbefund (bilaterale meist milchglasartige peripher und basal betonte Verdichtungen) aber ausreichend.

In begründeten Fällen (s. oben), bei schweren Verläufen oder zur besseren Differenzierung von Alternativdiagnosen bzw. Komplikationen ist eine CT des Thorax indiziert [60]. COVID-19-typische CT-Befunde sind bilaterale, multifokale, tendenziell peripher/subpleural und dorsobasal betonte Milchglasveränderungen mit oder ohne Konsolidierungsareale. Im Verlauf können die Konsolidierungen zunehmen und auch ein „crazy paving“-Muster auftreten. Sensitivität, Spezifität und negativ und positiv prädiktive Werte der CT-Veränderungen wurden in einer größeren Studie mit 97, 25, 65 und 83 % beschrieben [61]. Somit kann eine SARS-CoV-2-CAP mittels CT sensitiv erfasst werden, die radiologischen Veränderungen können aber auch durch andere Infektionen oder Erkrankungen bedingt sein.

#### Spezifische Therapie der SARS-CoV-2-CAP

Zunächst sollte die Therapie einer SARS-CoV-2-CAP wie auch bei anderen bakteriellen oder viralen Pneumonien leitliniengerecht erfolgen (s. oben).

Viel diskutiert werden aktuell noch nicht ausreichend validierte antivirale und antiinflammatorische Therapieansätze (u. a. Remdesivir, Chloroquin, Hydroxychloroquin, Tocilizumab, rekombinantes Angiotensin Converting Enzyme 2). Sie sollten daher in der klinischen Routine nicht als Standardtherapie zum Einsatz kommen. Ihre Wirksamkeit und Verträglichkeit ist entsprechend den WHO-Empfehlungen zunächst in klinischen Studien, wenn möglich randomisiert und kontrolliert („randomised controlled trial“, RCT), zu prüfen [62, 63]. Sobald die Ergebnisse von RCT vorliegen, sind diese im Therapiemanagement zu berücksichtigen. Bis dahin sollten experimentelle Therapien außerhalb von klinischen Studien nur gut begründet und in ausgewählten Einzelfällen („compassionate use“) in Erwägung gezogen werden. Sie sollten nicht unkritisch und unter Berücksichtigung potenziell schädigender Nebenwirkungen verwendet und dann möglichst in Registern erfasst werden [64].

Die FDA hat am 28.03.2020 zur Behandlung von COVID-19 eine „Emergency Use Authorization“ für Chloroquin und Hydroxychloroquin (per os) ausgesprochen [65]. Die FDA weist darauf hin, dass zur Wirksamkeit nur in-vitro bzw. anekdotische klinische Daten und Fallserien vorliegen und prinzipiell Chloroquin bzw. Hydroxychloroquin weiter in RCT geprüft werden sollten. Trotzdem erlaubt die FDA in den USA ab sofort die Verwendung von Chloroquin und Hydroxychloroquin für hospitalisierte COVID-19-Patienten (Körpergewicht >50 kg) auch außerhalb von Studien. Von der EMA sind derzeit weder Chloroquin bzw. Hydroxychloroquin noch eine andere spezifische SARS-CoV-2-Therapie oder Impfung zugelassen.

#### Therapie mit systemischen Steroiden

Bis auf wenige Ausnahmen zeigt eine Vielzahl von Studien und Metaanalysen keinen Nutzen oder sogar eine erhöhte Letalität von systemischen Steroiden bei svCAP bzw. beim viralen „acute respiratory distress syndrome“ (vARDS; [66–68]). Dementsprechend wird evidenzbasiert von einem routinemäßigen Einsatz systemischer Steroide zur Behandlung von svCAP/vARDS inkl. COVID-19 abgeraten [62].

In bestimmten Ausnahmen können systemische Steroide jedoch auch bei viraler CAP erwogen werden:

- Hydrocortison ist bei therapierefraktärem Schock mit massiver hämodynamischer Instabilität entsprechend den Sepsis-Leitlinien indiziert [69, 70].
- Schwere COPD-Exazerbation: 0,5 mg Prednisolon/kg/Tag für 5–7 Tage, dann Stopp.
- Schwere Asthma-Exazerbation: 0,5 mg Prednisolon/kg/Tag für höchstens 7 Tage, dann über weitere 7 Tage ausschleichend.
- Im Verlauf einer svCAP können bei V. a. organisierende Pneumonie, V. a. postpneumonische interstitielle Pneumonie, V. a. hämophagozytische Lymphohistiozytose oder V. a. Exazerbation einer vorbestehenden Lungenfibrose systemische Steroide im Einzelfall erwogen werden.

#### Pneumologische Intensivmedizin

Intensiv- und beatmungspflichtige Patienten sollten entsprechend den allgemeingültigen nationalen und internationalen Empfehlungen behandelt werden. So ist bei der meist vorherrschenden schweren Oxygenierungsstörung eine Eskalation von Beatmungsmaske mit Reservoir („non-rebreather mask“) über nasale High-flow-Sauerstoffsysteme („high flow nasal oxygenation“, HFNO) zur nichtinvasiven Beatmung („non-invasive ventilation“, NIV) empfohlen. International wird in allen intensivmedizinischen Empfehlungen besonderes Augenmerk auf den Schutz der Behandler gelegt, etwa bei Maßnahmen wie Intubation, NIV, HFNO, Bronchoskopie oder Feuchtvernebelung [69, 71, 72]. Eine Aerosolproduktion ist bei Sauerstofftherapie, HFNO, Feuchtvernebelung und NIV mit Non-Vented-Systemen wahrscheinlich nicht wesentlich erhöht und ein relevant gesteigertes Risiko für das Personal zunächst nicht anzunehmen. Bei Intubation, Bronchoskopie, endotrachealem Absaugen und Verwendung von Vented-Systemen bzw. bei Fehlen eines Virenfilters im Exspirationsschenkel von Beatmungssystemen ist dagegen ein erhöhtes Risiko für das Personal häufig nachweisbar. Einen guten Überblick über die Aerosolproduktion und das daraus entstehende Risiko für Behandler bietet das Positionspapier der DGP [73].

Wenn verfügbar, sollten HFNO und NIV von COVID-19-Patienten in Unterdruckzimmern stattfinden. In der klinischen Realität ist in Österreich die Zahl an Unterdruckzimmern jedoch limitiert, und HFNO und NIV sind auch in anderen Räumlichkeiten vertretbar. Die Personenschutzmaßnahmen müssen jedoch konsequent eingehalten werden.

Da die Bildung von Aerosolen bei steigender HFNO-Flussrate ansteigt, sollte die Flussrate so niedrig wie gerade nötig eingestellt werden und eine Mund-Nasen-Maske oder FFP1-Maske zur Reduktion der Aerosolfreisetzung auf das Gesicht des Patienten appliziert werden. Prinzipiell sollte bei deutlich erhöhtem oder rasch progredientem Sauerstoffbedarf eine intensivierte Überwachung gewährleistet sein, da ein akutes respiratorisches Versagen mit sofortiger Intubationsnotwendigkeit ohne zeitliche Verzögerung erkannt werden muss.

Unabhängig von der Art der Beatmung wird die Anwendung eines Respirators mit Zweischlauchsystem und Bakterien‑/Virenfilter am Exspirationsteil empfohlen. Eine Beatmung mit Einschlauchsystem und Leckage-Maske (Vented-System) ist aufgrund der Aerosolbildung zu unterlassen. Beatmungsmaschinen für die außerklinische Beatmung inklusive OSAS-Therapie sollten daher bei SARS-CoV-2-positiven Patienten im stationären Setting nicht angewendet, sondern durch geeignete Ventilatoren oder eine entsprechende Maskenkonstruktion mit Filter am Ausatemventil ersetzt werden. Warmluftbefeuchter bei Heimventilatoren sollten nicht angewendet werden [74]. Sollten im Krisenfall nur Beatmungsgeräte mit Einschlauchsystem und distaler Flow-Messung (beispielsweise Respironics V60®, Draeger Oxylog®) zur Verfügung stehen, muss patientennahe ein Filter angebracht werden, wobei der dadurch erhöhte Atemwegswiderstand zu berücksichtigen ist. Bei Verwendung eines CPAP-Helms ist am Exspirationsteil ein Filter anzubringen.

Zur Intubation wird die Videolaryngoskopie und die „rapid sequence induction“ mit voller Relaxierung empfohlen, um eine Aerosolbildung, einen möglichen Hustenstoß des Patienten und eine Annäherung des Airway-Operators an den Patientenkopf möglichst zu verhindern. Feuchtvernebelungen sollten zugunsten der Verwendung von Dosieraerosolen unterbleiben.

Entsprechend dem Schweregrad der Oxygenierungsstörung besteht vielfach die Empfehlung zur Intubation und invasiven Beatmung bei einem Oxygenierungsindex (P_a_O_2_/F_i_O_2_) ≤200 [72]. Ob in diesem Fall alternativ eine nichtinvasive Beatmung weiterhin durchführbar ist, muss für jeden Patienten individuell beurteilt werden. Abhängig von pulmonalen Vorerkrankungen, dem Erfolg der NIV, dem klinischen Zustandsbild insbesondere in Hinblick auf die Belastung der Atemmuskulatur, der Kooperation des Patienten und auch von konsequenten Schutzmaßnahmen für das medizinische Personal ist speziell die Erfahrung des Anwenders mit NIV wesentlich. Liegt bereits ein ARDS vor, sollte den gängigen Empfehlungen entsprechend bei fehlender Besserung unter NIV mit einer Intubation nicht zu lange zugewartet werden.

**Figure 4:**
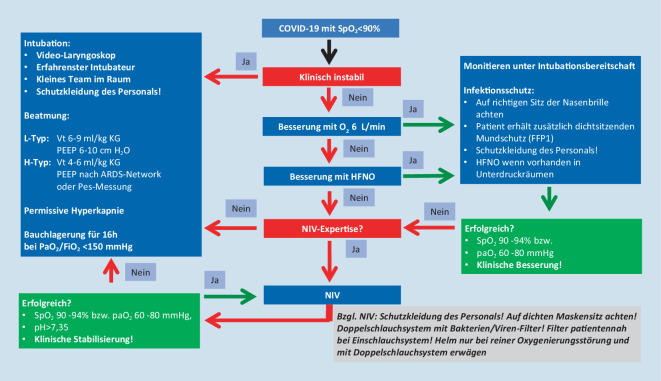

Aktuell unterscheidet man zwei Phänotypen der COVID-19-Lungenerkrankung (Abb. 4): Der L‑Typ („low elastance“) zeichnet sich durch gute Compliance, schlechtes Ansprechen auf Rekrutierungsmanöver und Verschlechterung bei Anwendung eines allzu hohen PEEP (>10 cm H_2_O) aus. Der oft schweren Oxygenierungsstörung liegen hier in erster Linie eine Vasoplegie mit veränderter Ventilations-Perfusions-Ratio sowie mikrothrombotische Ereignisse zugrunde.

Beim L‑Typ sind die O_2_/HFNO-Anwendung, eine NIV oder eine invasive Beatmung mit niedrigerem PEEP (6–10 cm H_2_O) aufgrund besserer kardialer Verträglichkeit sowie die Bauchlagerung meist effektiv. Höhere Atemzugvolumina werden gut und ohne Lungenschädigung („ventilator-induced lung injury“, VILI) vertragen.

Beim H‑Typ („high elastance“) liegen eine schlechtere Compliance (<40 ml/cm H_2_O), höherer Shunt, höhere rechtskardiale Drücke und besseres Ansprechen auf Rekrutierungsmaßnahmen vor, hier handelt es sich im Wesentlichen um das Vollbild eines schweren ARDS. Zielführend ist hier eine mechanische Beatmung mit eher hohem PEEP (invasiv meist >15 cm H_2_O), jedoch oft niedrigen Plateau-Drücken ausreichend. Es ist davon auszugehen, dass auch COVID-19-ARDS-Patienten von der Bauchlagerung („prone positioning“) nach dem ProSEVA-Protokoll deutlich profitieren [62, 75]. Rekrutierungsmanöver (Lachmann-Manöver) können beim H‑Typ versucht werden [76].

Ein Übergang vom L‑Typ in den H‑Typ ist möglich und kann frühzeitig an einer erhöhten Atemanstrengung (Ösophagusmanometrie, ZVD-Änderung, Beurteilung der „work of breathing“) erkannt werden.

Aus bisheriger Erfahrung und Obduktionsberichten zufolge wird Euvolämie empfohlen, da Überwässerung die respiratorische Situation überproportional verschlechtert.

Für die Anwendung oben bereits erwähnter experimenteller COVID-19-Therapien gibt es auch für intensivpflichtige Patienten derzeit keine überzeugende Evidenz. Nach dem Prinzip „primum nil nocere“ ist eine regelhafte Anwendung nicht ausreichend validierter und nicht zugelassener Medikamente unverändert nur in klinischen Studien bzw. in „compassionate use“-Programmen zu befürworten. Potenzielle Nebenwirkungen und mögliche Interaktionen mit typischen intensivmedizinischen Medikamenten sind zusätzlich zu bedenken [77]. Ebenso ist die Evidenz für eine supportive Therapie mit Zink, Ascorbinsäure oder Selen unzureichend.

Die Leitlinien der WHO zur Behandlung von COVID-19 umfassen das Thema Intensivmedizin und wir empfehlen die entsprechenden regelmäßigen Updates zu verfolgen und zu berücksichtigen [62].

Mikrozirkulationsstörungen auf thrombotischer Grundlage werden angenommen, und eine pharmakologische Thromboseprophylaxe ist unter Nutzen-Risiko-Abwägung auch bei der häufig auftretenden (moderaten) Thrombozytopenie jedenfalls indiziert [78, 79].

Ähnlich wie auch bei anderen schweren Infektionen kann sich bei COVID-19-ARDS Patienten eine Form der sekundären hämophagozytischen Lymphohistiozytose (sHLH) entwickeln. Es sollte daher auf Zeichen einer massiven hyperinflammatorischen Reaktion geachtet werden. Spezifische und ausreichend evaluierte diagnostische Kriterien für eine COVID-19-sHLH liegen noch nicht vor [80, 81]. Die sHLH-Diagnose und Klassifizierung orientierte sich bisher am praxisorientierten und auch evaluierten HScore [82, 83]. Ein frei verfügbarer Rechner hierfür findet sich unter http://saintantoine.aphp.fr/score/. Für die Therapie eines sHLH gibt es keinen Goldstandard. Die Evidenz beruht überwiegend auf Fallserien und es fehlen RCT. Wie auch bei anderen nicht COVID-19-assoziierten sHLH können daher im Einzelfall vor allem systemische Kortikosteroide, aber auch Cyclosporin, intravenöse Immunglobuline, Anakinra, Tocilizumab und andere Therapien in Erwägung gezogen werden [84].

#### Expositionsrisiko durch inhalative Therapien

Bei jeder Form von inhalativen oder atmungsunterstützenden Therapien (Feuchtvernebler, O_2_ über Nasenbrille/Maske, HFNO, NIV) ist bei COVID-19 mit Aerosolbildung und somit einem gesteigerten Infektionsrisiko für Gesundheitspersonal und Patienten zu rechnen (s. auch Abschnitt „Kardiorespiratorische Atemphysiotherapie“; [85]). Die entsprechenden Therapieformen sollten daher nur indikationsgerecht eingesetzt und unter Beachtung der möglichen Kontamination der Umgebung durch Aerosole eher restriktiv verwendet oder vermieden werden. Inhalative Bronchodilatatoren oder Kortikosteroide sollten vorzugsweise in Form von Trockenpulverinhalatoren oder (auch bei nichtinvasiver oder invasiver Beatmung) Dosieraerosolen zur Anwendung kommen [86]. Weitere Details finden sich in den Abschnitten „Pneumologische Intensivmedizin“ und „Kardiorespiratorische Physiotherapie“.

#### Hospitalisierte COVID-19-Patienten mit schlafbezogenen Atemstörungen

Erkrankt ein häuslich mit „positive airway pressure“ (PAP) behandelter Patient mit vorbekannter schlafbezogener Atemstörung an COVID-19, ist derzeit in Analogie zur NIV und Inhalationstherapie (s. oben) davon auszugehen, dass bei Nutzung dieser Therapie die Virusübertragung an die Umgebung gesteigert wird. In diesem Fall muss eine individuelle Risiko-Nutzen-Abwägung erfolgen. Wenn möglich, sollte die PAP aber unter Einhaltung strenger Hygiene- und Isolationsmaßnahmen weiterverwendet werden. Nach bisheriger Evidenz kommt es durch PAP zu keiner Verschlechterung einer COVID-19-Infektion.

Bei Verwendung von Einschlauchsystemen und „Vented“-Masken wird zum Schutz der Behandler empfohlen, den Warmluftbefeuchter nach Möglichkeit nicht zu verwenden und auf „Non-Vented“-Masken mit speziellem Ausatemventil und Filter zu wechseln. Wenn verfügbar, kann alternativ auch auf ein Zweischlauchsystem umgestellt werden.

#### Bronchoskopie bei COVID-19-Patienten

Es wird empfohlen, keine Bronchoskopie zum Ausschluss oder Nachweis von COVID-19 durchzuführen (fehlende therapeutische Konsequenz, unnötiges Risiko für das Personal, mögliches Risiko der klinischen Verschlechterung durch die Bronchoskopie). In speziellen Situationen kann eine Bronchoskopie jedoch auch bei bestätigten oder suspizierten COVID-19-Patienten indiziert sein (z. B. bei Immunsuppression zum Ausschluss einer *Pneumocystis*-Pneumonie).

Im Rahmen einer Bronchoskopie besteht prinzipiell das Risiko einer Aerosolbildung und somit ein deutlich erhöhtes SARS-CoV-2-Infektionsrisiko für das bei der Untersuchung anwesende Personal. Eine Bronchoskopie beim intubierten Patienten hat wahrscheinlich ein geringeres Transmissionsrisiko.

In Anlehnung an internationale Empfehlungen ist für die Zeit der COVID-19-Pandemie bei Verdacht auf oder gesicherter SARS-CoV-2-Infektion Folgendes zu beachten [87–89]:

- Sehr kritische Indikationsstellung für eine Bronchoskopie.
- Primäre Nutzung anderer sensitiver diagnostischer Verfahren (z. B. Gewinnung von Trachealsekret via geschlossenem Absaugsystem für mikrobiologische Diagnostik inkl. SARS-CoV-2-PCR).
- Eine Indikation für eine Bronchoskopie besteht für notfallmäßige Eingriffe (beispielsweise bedrohliche Hämoptoe, höhergradige Atemwegsstenose oder Fremdkörperaspiration), oder wenn durch die Bronchoskopie eine alternative Diagnose verifiziert werden kann, die zu einer signifikanten Änderung des therapeutischen Managements führen würde.
- Reduktion der Mitarbeiter (Bronchoskopiker, Bronchoskopieassistenz, ggf. Anästhesieteam) auf ein Kernteam. Keine Studierenden, Aus- oder Weiterzubildenden im Untersuchungsraum.
- Strenger persönlicher Schutz für das gesamte Team (Einmalschutzkittel, Einmalhandschuhe, FFP3-Maske, Schutzbrille/Schutzvisier, Haarschutz). Striktes Achten auf korrektes An- und Ablegen der Schutzkleidung.
- Starre Bronchoskopien mit Jet-Ventilation sollten, soweit medizinisch vertretbar, nicht durchgeführt werden. Wenn eine starre Bronchoskopie unvermeidbar ist, dann nur bei einem intubierten Patienten mit konventioneller Beatmung und Reduktion des Austritts von Aerosol, z. B. mittels FLUVOG-Aufsatz.
- Bei Durchführung einer Lavage fraktioniertes Vorgehen (jeweils 10 ml NaCl 0,9 %; zur Verminderung des Transmissionsrisikos Abklemmen des Saugers nach Probengewinnung bzw. vor Diskonnektion).
- Bei zeitnaher und validierter Aufbereitung der Bronchoskope besteht kein Anhalt dafür, dass der Aufbereitungsprozess zur Desinfektion der Geräte bei SARS-CoV‑2 geändert werden muss.

Routine-Bronchoskopien bei primär Nicht-COVID-19-Patienten (z. B. zur Abklärung von pulmonalen Rundherden/Raumforderungen oder interstitiellen Lungenerkrankungen) sollten während der aktuellen Pandemie-Situation ebenfalls nur bei strenger Indikationsstellung, unter erhöhten persönlichen Schutzmaßnahmen (inklusive Verwendung von FFP2- oder FFP3-Masken) und auch verschärften hygienetechnischen Bronchoskopie-Modifikationen durchgeführt werden.

#### Therapieziele, Therapiebegrenzungen und Therapierückzug bei COVID-19

**Figure 5:**
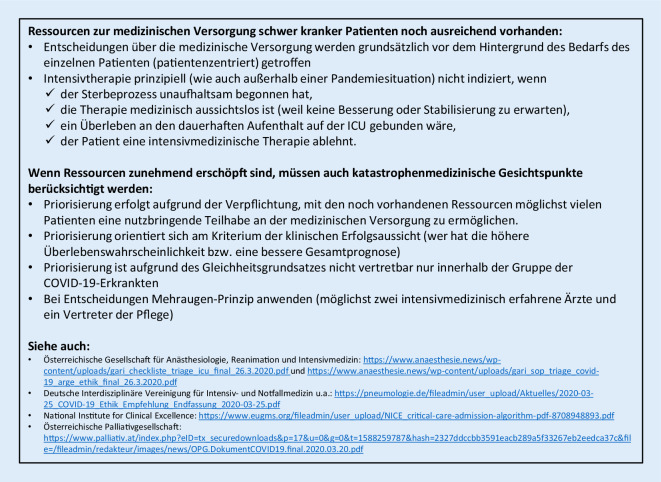

Die ethischen Grundsätze der intensivmedizinischen und palliativmedizinischen Versorgung gelten für COVID-19-Patienten gleichermaßen. Da es aber in mehreren Ländern zu einer vollständigen Ausschöpfung sogar vermehrter intensivmedizinischer Ressourcen gekommen ist, wurden in Österreich Leitlinien für die Allokation von Intensivbetten, für eine Triage und für die palliativmedizinische Behandlung erstellt [90, 91]. So sollen anhand des vorbestehenden Gesundheitszustands und der Schwere der Erkrankung sowie unter Berücksichtigung des Patientenwillens Kapazitäten vordringlich für Patienten freigehalten werden, für die eine höhere Überlebenswahrscheinlichkeit vorhergesagt wird [92]. Dies ist nicht nur aufgrund fehlender validierter, prädiktiver Scores für COVID-19 ein schwieriges Unterfangen, sondern blendet auch das Problem aus, dass Patienten ohne SARS-CoV-2-Infektion oder solche mit klinisch stummer Infektion aus anderen Gründen einer intensivmedizinischen Behandlung bedürfen können (z. B. COPD-Exazerbation, Myokardinfarkt, Polytrauma …; Abb. 5). Die deutschen und britischen Fachgesellschaften haben ebenfalls Handlungsempfehlungen zum Thema „klinisch-ethische Entscheidungsfindung“ erarbeitet [93, 94].

## Allgemeines Management von Patienten mit chronischen Lungenerkrankungen während der COVID-19-Pandemie

### Präventivmaßnahmen zur Vermeidung von COVID‑19 und/oder schwerer Verläufe (Empfehlungen für Patienten mit vorbestehenden Grunderkrankungen)

**Figure 6:**
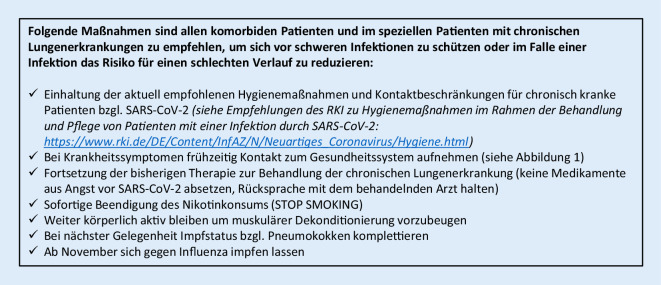

Patienten mit chronischen Lungenerkrankungen haben die Möglichkeit, sich vor schweren Infektionen zu schützen oder im Fall einer Infektion das Risiko für einen schlechten Verlauf zu reduzieren. Folgende Maßnahmen sind von besonderer Bedeutung (Abb. 6):

- Einhaltung der aktuell empfohlenen Hygienemaßnahmen und Kontaktbeschränkungen für chronisch kranke Patienten
- Bei Krankheitssymptomen frühzeitige Kontaktaufnahme mit dem Gesundheitssystem (Abb. 1)
- Fortsetzung der bisherigen Therapie zur Behandlung der chronischen Lungenerkrankung (kein Absetzen von Medikamenten aus Angst vor SARS-CoV‑2, Rücksprache mit dem behandelnden Arzt)
- Sofortige Beendigung des Nikotinkonsums, da Rauchen das Risiko an COVID-19 zu versterben deutlich erhöht [95]
- Körperliche Aktivität, um einer muskulären Dekonditionierung vorzubeugen
- Bei nächster Gelegenheit Komplettierung des Impfstatus bzgl. Pneumokokken
- Impfung gegen Influenza ab November 2020

Zusätzlich kann in der aktuellen COVID-19-Pandemie erwogen werden, dass besonders gefährdete Patientengruppen zeitweilig von beruflichen Tätigkeiten im öffentlichen Raum freigestellt werden sollten. Das Ziel dieser Maßnahme wäre, die Zahl der in den nächsten kritischen Wochen akut schwer erkrankten Personen zu reduzieren, um das Gesundheitssystem zu entlasten. Anhand der bisherigen COVID-19-Daten und der evidenzbasierten klinischen Erfahrung mit anderen akuten respiratorischen Virusinfektionen kann dies orientierend für folgende Patienten mit chronischen Lungenerkrankungen erwogen werden:

- Alter >65 Jahre und schwere Lungenerkrankung jeglicher Art
- Alter ≤65 Jahre mit
  - pO_2_ <65 mm Hg unter Raumluft oder
  - Langzeitsauerstofftherapie (LTOT) oder
  - FEV1 <70 % Sollwert oder
  - FVC <70 % Sollwert oder
  - Diffusionskapazität <70 % Sollwert oder
  - zystischer Fibrose oder
  - aktiver Krebserkrankung oder
  - systemischer immunsuppressiver Therapie bzw. kongenitaler Immundefizienz

Patienten ≤65 Jahre mit einer nur leichten COPD oder einem leichten Asthma und gut kontrollierter Symptomatik haben kein übermäßig erhöhtes Risiko, schwer an SARS-CoV‑2 zu erkranken. Eine generelle Arbeitsfreistellung ist bei diesen Patienten nicht erforderlich.

#### Influenza-Impfung

In nur knapp mehr als einem halben Jahr wird die Influenza-Saison 2020/2021 beginnen. Zu diesem Zeitpunkt wird SARS-CoV‑2 das Gesundheitssystem vermutlich noch immer belasten. In Anbetracht der bekannten jährlichen hohen Influenza-Erkrankungs- und Letalitätsrate (s. oben) und der chronisch niedrigen Influenza-Durchimpfungsrate (Allgemeinbevölkerung <10 % und Risikogruppen <25 %) ist somit ab Dezember 2020 mit einer COVID-19 plus Influenza Doppelbelastung zu rechnen. Wir empfehlen daher bereits jetzt, die notwendigen effektiven Präventivmaßnahmen für die Influenza-Saison 2020/2021 in die Wege zu leiten. Dazu gehört aus Sicht der Österreichischen Gesellschaft für Pneumologie und der „Influenza-Task-Force Österreich“ auch, dass die Influenzaschutzimpfung in Österreich (wie in den meisten europäischen Ländern üblich) deutlich niedrigschwelliger und kostenfrei angeboten werden sollte.

### Asthma und COPD

#### Asthma

Bei gut kontrolliertem Asthma besteht grundsätzlich kein erhöhtes Risiko für einen komplikationsbehafteten COVID-19-Verlauf; anders verhält es sich bei schlecht kontrolliertem Asthma. Es ist daher besonders wichtig, dass Asthmapatienten ihre Medikamente, die zu einer guten Krankheitskontrolle beitragen können, jetzt besonders konsequent einnehmen. Das gilt insbesondere auch für inhalative Kortisonpräparate, die eine gute Kontrolle dieser chronisch-entzündlichen Erkrankung ermöglichen. Auch die Therapie mit Biologicals (wie Omalizumab, Mepolizumab, Reslizumab, Benralizumab und Dupilumab) sollte unverändert fortgesetzt werden. Eine negative Beeinflussung der Immunabwehr gegen SARS-CoV‑2 ist durch die Biologicals nach jetzigem Wissenstand nicht zu erwarten, während dies bei oralen Kortikosteroiden (als therapeutische Alternative) zu erwarten ist.

Keinesfalls sollten die Medikamente unter der Annahme, dass sie das Immunsystem schwächen könnten, abgesetzt werden, denn ein gut kontrolliertes Asthma ist die beste Voraussetzung für einen milden Verlauf einer SARS-CoV-2-Infektion. Daneben sollten Patienten mit Asthma genau auf markante Änderungen ihrer Symptomatik achten, insbesondere auf plötzlich zunehmende Atemnot, neu auftretenden Husten sowie Fieber. Während die Symptome Atemnot und Husten bei Patienten mit Asthma gängig sind, deutet das Symptom Fieber durchaus auf eine Infektion hin und sollte ernst genommen und abgeklärt werden.

#### COPD

Auch COPD-Patienten sollten ihre regelmäßig verwendete Therapie gerade jetzt möglichst konsequent durchführen, um die gefürchteten akuten Verschlechterungen der COPD, sog. Exazerbationen, möglichst zu verhindern. Kommt es in der gegenwärtigen Situation zu einer Exazerbation, die u. U. einen Arztbesuch oder Krankenhausaufenthalt erfordert (Abb. 1), können Patienten die wichtigsten zum Schutz vor einer SARS-CoV-2-Infektion empfohlenen Maßnahmen nicht einhalten, nämlich zu Hause zu bleiben und von anderen Personen Abstand zu halten. Generell trägt die regelmäßig verwendete Medikation auch bei COPD zu einer guten Kontrolle der Erkrankung bei, weshalb eine hohe Therapieadhärenz gerade in Zeiten der SARS-CoV-2-Pandemie von Vorteil ist. Da COPD typischerweise von Atemnot und Husten begleitet ist, lässt sich eine SARS-CoV-2-Infektion bei diesen Patienten am ehesten über plötzlich stark zunehmende Atemnot und Fieber erkennen (Abb. 1). Dem Fieber könnte eine Exazerbation, aber eben auch eine SARS-CoV-2-Infektion zugrunde liegen. Während systemische Glukokortikoide bei COVID-19 derzeit nicht empfohlen sind, ist ihr Einsatz zur Behandlung der gewöhnlichen COPD-Exazerbation durchaus gerechtfertigt.

#### Asthma- und COPD-Patienten mit wahrscheinlicher oder bestätigter SARS-CoV-2-Infektion

Bei bestätigter SARS-CoV-2-Infektion haben auch Patienten mit einer chronischen Atemwegserkrankung durchaus Chancen auf einen milden Verlauf, der in häuslicher Isolierung bewältigt werden kann. Empfohlen sind hier fiebersenkende Maßnahmen, um allzu hohes, den Kreislauf belastendes Fieber zu vermeiden, sowie ausreichende Flüssigkeitszufuhr. Die Sauerstoffsättigung kontrollieren viele COPD-Patienten auch in stabilen Phasen ihrer Erkrankung selbstständig mit einem Fingerpulsoximeter. Fällt die Sauerstoffsättigung unter den sonst üblichen Bereich, sollte ärztliche Hilfe in Anspruch genommen werden. Fehlt die Möglichkeit, die Sauerstoffsättigung zu Hause zu kontrollieren, sollte die Atemsituation genau beobachtet werden. Zunehmende Atemnot in Ruhe oder bei minimaler körperlicher Belastung ist ein Grund, ärztliche Hilfe zu suchen. Patienten mit Asthma sollten in gewohnter Weise ihre Symptomatik und Peak-Flow-Werte dokumentieren. Markante Veränderungen bedürfen ärztlicher Abklärung. Prinzipiell kann sich die Atemsymptomatik chronisch Lungenkranker durch COVID-19 vorübergehend verschlechtern, im Regelfall heilt die Erkrankung jedoch ohne Folgen ab und eine vollständige Erholung der Lungenfunktion ist zu erwarten.

### Lungenkarzinom

Derzeit gibt es keine Evidenz dafür, eine antitumoröse Therapie wie Chemotherapie und/oder Immuntherapie abzubrechen oder zu pausieren. Diagnostik und Therapie sollten nach bisherigen Standards fortgeführt werden. Die Entscheidungen sind aber immer individuell zwischen Arzt und Patient abzuwägen. Für weitere Details verweisen wir auf die aktuellen Empfehlungen der ASCO, der ESMO und der DGHO/ÖGHO [96–98].

### Zystische Fibrose (CF; Mukoviszidose)

Erwachsene Patienten mit zystischer Fibrose zählen zur Risikogruppe für eine möglicherweise schwer verlaufende Infektion mit SARS-CoV‑2. Neben der Einhaltung der allgemein gültigen Präventionsmaßnahmen (Händehygiene, Abstandsregel) sollten CF-Patienten nach Möglichkeit zu Hause bleiben und keine sozialen Kontakte im beruflichen oder sonstigen sozialen Umfeld suchen. Wenn möglich, sollten während der Pandemie auch Lebensmitteleinkäufe und die Besorgung von Medikamenten oder Atemphysiotherapiehilfsmitteln durch dritte Personen erfolgen.

Weiters ist es betreffend Routinevorstellungen in den nächsten Wochen notwendig, Kontakt mit dem jeweilig betreuenden Zentrum aufzunehmen, um zu klären, welche Untersuchungen verschoben bzw. welche Ambulanzbesuche unter speziellen Bedingungen in der jeweiligen Einheit stattfinden sollen, und ob im individuellen Fall auch telefonische Anweisungen statt eines Besuchs erfolgen können.

CF-Zentren sind bemüht, nicht zwingend notwendige Ambulanzbesuche in den nächsten Wochen auszusetzen, wenn dadurch keine Nachteile für die betreuten Patienten entstehen. Die üblichen therapeutischen Maßnahmen wie Atemphysiotherapie, medikamentöse und Ernährungstherapie sollten besonders sorgfältig fortgesetzt werden. Bei klinischer Verschlechterung, insbesondere bei Auftreten von Fieber und vermehrtem Husten mit oder ohne Atemnot ist, neben den sonst zu Hause zur Verfügung stehenden Erstmaßnahmen wie Einnahme von Fieber senkenden Medikamenten und Anwendung der üblichen inhalativen Medikation, eine telefonische Kontaktaufnahme mit einem Arzt bzw. dem betreuenden Zentrum sinnvoll, in jedem Fall bei einer bereits geplanten Vorstellung im Zentrum zur weiteren Behandlung.

Klinisch indizierte, stationäre i.v.-Antibiotikatherapien sollten jedenfalls durchgeführt werden. Bei einer notwendigen stationären Behandlung einer SARS-CoV-2-Erkrankung wird in jedem Fall, neben der den Leitlinien der medizinischen Fachgesellschaften entsprechenden Behandlung, auch eine spezifische, dem jeweiligen Keimspektrum angepasste Antibiotikatherapie einzuleiten sein.

Ein kürzlich veröffentlichter Artikel berichtet von 10 SARS-CoV-2-infizierten CF-Patienten in der Lombardei (von insgesamt 42.161 Infizierten in der Lombardei und 101.739 in ganz Italien am 31.03.2020). Die Übertragung der Infektion erfolgte jeweils durch einen Familienangehörigen. Darüber hinaus wurden fünf Patienten in Frankreich, sieben in Großbritannien, fünf in Deutschland und drei (darunter ein Transplantierter) in Spanien berichtet (ausschließlich Erwachsene; [99]). Bei diesen wenigen Patienten führte die SARS-CoV-2-Infektion zu keiner erkennbaren Verschlechterung der Grunderkrankung. Die CF-Zentren sind aufgefordert, mit SARS-CoV‑2 infizierte Patienten an das europäische CF-Register zu melden (servicedesk@ecfregistry.eu).

### Interstitielle Lungenerkrankungen

Patienten mit einer interstitiellen Lungenerkrankung (ILE) gehören aufgrund der strukturellen Lungenveränderungen, immunsuppressiven Therapie, Diffusionsstörung mit häufig bereits bestehendem O_2_-Bedarf und fortgeschrittenem Alter zur COVID-19-Risikogruppe. Um das Infektionsrisiko zu minimieren, sollten ILE-Patienten die soziale Isolation und alle empfohlenen Schutzmaßnahmen rigoros durchführen. Dabei ist eine Unterstützung durch Familienmitglieder, Nachbarn und Hilfsorganisationen essenziell, um die Versorgung mit notwendigen Lebensmitteln und Medikamenten zu organisieren; gleichzeitig sollte aber auf den direkten Kontakt mit Personen, die nicht im gleichen Haushalt leben, möglichst verzichtet werden.

Die Terminvergabe für ILE-Ambulanzen sollte optimiert werden, um längere Wartezeiten und Patientenansammlungen zu vermeiden. Bei schriftlicher Einwilligung und technischer Bereitschaft können für routinemäßige Verlaufskontrollen auch Alternativen wie Videochats erwogen werden. Um den direkten Kontakt zwischen Ärzten und damit auch das Risiko einer Infektionsübertragung zu minimieren, sollten alternative (beispielsweise digitale) Kommunikationsformen auch für multidisziplinäre Falldiskussionen (ILE Board) in Betracht gezogen werden.

Für die rechtzeitige Diagnose einer SARS-CoV-2-Infektion ist es erforderlich, eine PCR-Testung bereits bei Auftreten neuer Krankheitserscheinungen durchzuführen. So kann frühzeitig eine andere Ursache für die Symptome oder eine akute Exazerbation entdeckt und eine entsprechende Behandlung ohne Verzögerung eingeleitet werden.

Viele ILE-Patienten haben eine immunsuppressive Therapie, daher ist im Fall einer Virusinfektion von schwereren Verläufen auszugehen. Eine antifibrotische Therapie bei fibrosierenden ILE und eine immunsuppressive Therapie bei inflammatorischen ILE sollte bei nicht an COVID-19 erkrankten ILE-Patienten nicht unterbrochen werden, um keine Exazerbation der ILE zu riskieren. Liegt eine bestätigte SARS-CoV-2-Infektion vor, muss individuell abgewogen werden, ob eine immunsuppressive Therapie reduziert oder passager abgesetzt werden soll.

Die Behandlung von Patienten mit einer fortgeschrittenen ILE und COVID-19 wird wahrscheinlich schwierige therapeutische Entscheidungen erfordern und ethische Bedenken hervorrufen. In diesem Sinne ist es erforderlich, mit den Betroffenen und deren Familien rechtzeitig über diese Probleme zu sprechen und, soweit möglich, einen Therapiehorizont festzulegen. Für Patienten mit fortgeschrittener ILE und COVID-19 sollten auch palliative Maßnahmen erwogen werden.

### Pulmonale Hypertonie

Patienten mit pulmonaler Hypertonie, im Speziellen mit pulmonalarterieller Hypertonie (PAH), zählen zu den Risikopatienten. Es gibt allerdings keine Daten darüber, wie sich der klinische Verlauf von COVID-19 bei Patienten mit PAH manifestiert. Es sind uns auch keine aktuellen Publikationen bekannt, die besondere Zusammenhänge zwischen dieser Viruserkrankung und pulmonalen Gefäßerkrankungen untersucht haben.

Wie bei anderen Lungenerkrankungen ist bei Patienten mit PAH die Infektprävention im multimodalen Therapieansatz generell wichtig, da in Abhängigkeit auch von der Schwere der Grunderkrankung bereits durch milde respiratorische Infekte passagere Erhöhungen der Druckbelastung des rechten Herzens bis zur klinischen Dekompensation beobachtet wurden. Eine Viruspneumonie durch SARS-CoV‑2 führt zu einer Verschlechterung der Oxygenierung, und die begleitende lokale und systemische Entzündungsreaktion lässt auch die Möglichkeit der Verschlechterung der Rechtsherzsituation vermuten. In einer Autopsie-Studie wurde dann auch eine Häufung von ausgeprägter Rechtsherzdilatation bei verstorbenen COVID-19-Patienten beschrieben [100].

Daraus abgeleitet sind die behördlich empfohlenen Maßnahmen zum Social Distancing insbesondere auch für Patienten mit pulmonalen Gefäßerkrankungen wichtig. Allerdings sollte es dadurch nicht zu einer verzögerten Diagnostik für Patienten kommen. Verdachtsfälle auf eine akute Lungenembolie sollten weiterhin leitliniengemäß und ohne Verzögerung abgeklärt und behandelt werden, sodass Patienten mit einer potenziell tödlichen akuten Erkrankung nicht zu Schaden kommen. Bei Patienten mit Verdacht auf eine schwere pulmonale Hypertonie sollte ebenfalls ohne Verzögerung eine Abklärung inklusive Rechtsherzkatheter erfolgen und eine leitliniengerechte Therapie eingeleitet werden.

Patienten unter PAH-Therapie sollen die allgemein empfohlenen Hygiene- und sonstigen Maßnahmen einhalten und bei Zeichen einer SARS-CoV-2-Infektion abhängig von der Schwere der Symptomatik den Hausarzt bzw. den niedergelassenen Facharzt oder Facharzt im Zentrum kontaktieren und frühzeitig mit einer antibiotischen Therapie beginnen.

Über die Notwendigkeit einer regelmäßigen ambulanten Kontrolle im PAH-Zentrum soll individuell entschieden werden. Patienten sollen bzgl. der spezifischen PAH-Medikation vorsorgen (Medikation für mindestens 8 Wochen) und ggf. bei Lieferengpässen rechtzeitig mit dem PAH-Zentrum Kontakt aufnehmen. Ein engmaschiger telefonischer Kontakt mit Patienten ist sinnvoll und sollte von den Zentren praktiziert werden.

### Pneumologische Rehabilitation und Rauchertherapie

Die Pensionsversicherungsanstalt (PVA) wird als kritische Infrastruktur der Republik Österreich eingestuft. Sie ist gesetzlich verpflichtet, den Betrieb, insbesondere auch der eigenen Rehabilitationszentren, aufrechtzuerhalten. Im Bereich der Gesundheitsleistungen werden derzeit in den stationären Rehabilitationszentren weiterhin jene Personen betreut, die nach akutmedizinischen Ereignissen oder Eingriffen dringend Versorgung und Rehabilitation brauchen. Gleichzeitig werden aktuell all jene Maßnahmen, die nicht dringend notwendig sind, zurückgefahren [101].

Die pneumologischen ambulanten und stationären Rehabilitationszentren haben aufgrund des Risikoprofils der betroffenen Patienten bis auf weiteres geschlossen. Die Strukturen in räumlicher und personeller Hinsicht wurden zur Versorgung von Patienten in Kooperation mit umliegenden Spitälern koordiniert.

Nach Beendigung der COVID-19-Pandemie erwarten die Rehabilitationszentren eine große Nachfrage an Rehabilitationsbehandlungen. Diese wird zum einen durch den Rückstau von verschobenen Rehabilitationsbehandlungen und zum anderen durch die große Zahl von COVID-19-Opfern bedingt sein. Dieser zu erwartende Bedarf sollte vorausschauend berücksichtigt werden.

### Schlafbezogene Atemstörungen

Entsprechend dem aktuellen Statement der Deutschen Gesellschaft für Schlafmedizin [102] schließen wir uns folgender Beurteilung an: Es gibt keine verlässlichen Informationen darüber, ob Schlafapnoepatienten ein erhöhtes Risiko für eine SARS-CoV-2-Infektion oder ein erhöhtes Risiko für einen schweren Verlauf haben. Viele Schlafapnoepatienten sind jedoch älter (>65 Jahre) und zusätzlich an typischen Begleit- oder Folgeerkrankungen der Schlafapnoe erkrankt (Lungen‑, Herz‑, Nieren‑, Lebererkrankungen, schwere Adipositas, Diabetes mellitus), die als Risikofaktoren für einen schweren COVID-19-Verlauf gelten. Neben den allgemeinen Empfehlungen der Gesundheitsbehörden können derzeit jedoch keine evidenzbasierten spezifischen Empfehlungen für asymptomatische Patienten unter PAP-Therapie ausgesprochen werden. Für die Reinigung/Desinfektion von CPAP-Geräten sind unverändert die Vorgaben des jeweiligen Herstellers ausschlaggebend.

Wegen der möglichen SARS-CoV-2-Umweltexposition über CPAP/APAP kann bei besorgten asymptomatischen Patienten oder Angehörigen die passagere Möglichkeit getrennter Schlafzimmer während der Pandemie diskutiert werden [103].

### Transplantation

Bisher wurden nur sehr wenige COVID-19-Fälle nach stattgehabter solider Organtransplantation veröffentlicht. Das Thema wird aber innerhalb der Transplantvereinigungen beobachtet und diskutiert [104]. Ähnlich wie in der Normalbevölkerung kann eine SARS-CoV-2-Infektion nach Transplantation sehr unterschiedlich verlaufen. Ein besonders erhöhtes Risiko für schwere Verläufe wurde für Transplantationspatienten noch nicht festgestellt [105].

Bei immunsupprimierten Patienten kann die Symptomatik atypisch mit gastrointestinalen Symptomen und Fieber beginnen; pulmonale Symptome treten später auf [106]. Somit sollte bei Transplantationspatienten auch bei extrapulmonalen Infekten an COVID-19 gedacht werden.

Die meisten Transplantationsprogramme wurden im Rahmen der COVID-19-Pandemie vorübergehend gestoppt. Nur „high urgent“ Fälle sollten derzeit transplantiert werden, und alle Spender und Empfänger werden auf SARS-CoV‑2 getestet. Das Standard Follow-up nach Transplantation wird vorübergehend minimiert, vor allem routinemäßige Krankenhausbesuche sollten reduziert und ggf. kostenlose telemedizinische Methoden verstärkt genutzt werden (z. B. www.daag.de).

Das Management von Transplantationspatienten mit einer SARS-CoV-2-Infektion ist noch nicht standardisiert. Bei asymptomatischen Patienten sollte keine Therapieänderung erfolgen. Bei Symptomen empfiehlt sich eine Pausierung von Mycophenolat oder Azathioprin; Calcineurin-Inhibitoren sollten reduziert werden. Ein ähnliches Vorgehen empfiehlt auch die Österreichische Gesellschaft für Nephrologie nach Nierentransplantation [107]. Ob eine Dosiserhöhung der Kortikosteroide bei Lungentransplantationspatienten sinnvoll ist, kann derzeit noch nicht beantwortet werden.

COVID-19-ARDS-Patienten mit refraktärem respiratorischem Mono-Organversagen (erfolgloses Weaning) können nach Abklingen der floriden Infektion in Abhängigkeit vom Alter, dem Allgemeinzustand und bei fehlenden relevanten Komorbiditäten für eine Lungentransplantation in Betracht gezogen werden [108].

### Kardiorespiratorische Physiotherapie

Wie bei den meisten viralen Pneumonien handelt es sich bei der SARS-CoV-2-CAP um eine interstitielle Pneumonie. Dementsprechend bildet sich bei der SARS-CoV-2-Infektion intraalveolär und -bronchial zunächst kein relevantes purulentes Sekret, und klinisch steht daher ein trockener, nichtproduktiver Husten im Vordergrund. In diesen Fällen sind atemphysiotherapeutische Maßnahmen nicht notwendig. Somit gilt: keine Atemphysiotherapie (APT) bei COVID-19-Patienten mit milden Symptomen!

Exsudative Konsolidierungen (z. B. durch sekundäre bakterielle Infektionen) und Hypersekretion mit Schwierigkeiten bei der Sekret-Entfernung können jedoch im Verlauf von COVID-19 auftreten oder bei bestimmten Vorerkrankungen von Anbeginn vorliegen. Dies betrifft beispielsweise Patienten mit zusätzlich schwerer obstruktiver Lungenerkrankung (COPD, Asthma), CF oder Bronchiektasen im Rahmen anderer Erkrankungen, aber auch Patienten mit neuromuskulären Erkrankungen oder Rückenmarksverletzungen. In diesen Situationen kann bei Vorliegen aktueller oder absehbarer Probleme bei der Sekret-Entfernung APT indiziert sein. Für jeden einzelnen Patienten muss individuell abgewogen werden, ob die Intervention das mögliche Infektionsrisiko für das Personal rechtfertigt (keine Durchführung unnötiger Therapien).

#### Physiotherapeutische Interventionen, die potenziell mit einer gesteigerten Virusexposition verbunden sind, inkludieren

- Inhalationstraining, Sekret fördernde Techniken und Sputum-Induktion
- Manuelle und maschinelle Hustenunterstützung (Cough-Assist)
- Sauerstofftherapie (auch mit Nasenbrille)
- Intermittent Positive Pressure Breathing (IPPB) und NIV
- Absaugen aus dem Luftweg
- Mobilisation und Training
- Versorgung tracheotomierter Patienten
- In- und exspiratorisches Muskeltraining

Jeder unnötige Kontakt (z. B. sonst übliche Routinebesuche) ist derzeit bei bestätigter oder suspekter SARS-CoV-2-Infektion zu unterlassen, und Assessments sollten nach Möglichkeit nicht mit direktem Körperkontakt durchgeführt werden (eventuell Telefonkontakt, Kommunikation von Informationen über das Pflegepersonal).

Alle atemphysiotherapeutischen Techniken, welche potenziell Husten stimulieren oder Sekret mobilisieren, erhöhen das Risiko einer Virusübertragung. Das Risiko einer Virusübertragung muss in jedem einzelnen Fall sehr sorgfältig abgewogen und entsprechende Sicherheitsmaßnahmen müssen getroffen werden.

#### Inhalationen

Flüssiginhalationen bei nichtintubierten Patienten mit COVID-19 mit Jet‑/Membran- oder Ultraschall-Verneblern werden primär nicht empfohlen, da es hierbei ähnlich wie bei NIV, HFNO oder Lungenfunktionsuntersuchungen zu einer erhöhten Virusfreisetzung in die Umgebungsluft kommen kann (Infektionsrisiko für medizinisches Personal). Stattdessen sollten Inhalationen mit einem Dosieraerosol in Kombination mit einer Vorschaltkammer bevorzugt werden. Wenn eine Flüssiginhalation unumgänglich ist, sollten dem Exspirationsventil ein Virenfilter vorgeschaltet und unbedingt geeignete Schutzmaßnahmen für das medizinische Personal getroffen werden.

#### Grundregeln für physiotherapeutische Interventionen an intubierten/tracheotomierten Patienten mit COVID-19 oder Verdacht auf COVID-19

- Absaugen bei intubierten/tracheotomierten Patienten nur mit geschlossenem Absaugsystem.
- Eine Diskonnektion des beatmeten Patienten vom Beatmungsgerät ist generell zu vermeiden – wenn unumgänglich, sollte dies nach Möglichkeit nur mit abgeklemmtem Tubus und deaktivierter Beatmungsmaschine durchgeführt werden.
- Das Ablassen des Cuffs einer Trachealkanüle, ebenso wie das Reinigen der Innenkanüle ist potenziell mit dem Risiko einer Virusübertragung durch die Luft verbunden.
- Inspiratorisches Muskeltraining, das Verwenden von Sprechventilen und Sprechtraining mit Trachealkanüle sollten erst nach dem Abklingen der akuten Infektion stattfinden, um das Risiko einer Virusübertragung zu minimieren.
- Immer Verwendung persönlicher Schutzausrüstung (Maske; Brille; langärmelige Schutzmäntel; Handschuhe; bei Personal mit Bart sollte die Gesichtsbehaarung soweit entfernt werden, dass ein ordnungsgemäßer Sitz der Gesichtsmaske möglich ist; bei Interventionen, welche die Viruslast im Raum erhöhen, ist ein Haarschutz zu tragen).

#### Allgemeine zusätzliche Empfehlungen für APT bei nichtintubierten Patienten

- Immer Verwendung persönlicher Schutzausrüstung (s. oben)
- Husten-Etikette einhalten (gilt für Personal und Patienten):
  - Während des Hustens und des Expektorierens den Kopf abwenden
  - Das Sekret in einem Taschentuch oder Behälter auffangen und gleich entsorgen – anschließend obligatorische Händedesinfektion
  - Bei geplanten Hustenmanövern: Mindestens 2 m Abstand und/oder aus der Hustenlinie gehen
- Physiotherapeutische Interventionen nur nach Anforderung/Rücksprache mit dem verantwortlichen Arzt
- Im Fall der Notwendigkeit einer physiotherapeutischen Intervention mit potenziellem Risiko einer Virusübertragung:
  - Durchführung im Ein-Bett-Zimmer bei geschlossener Türe
  - Minimum an Personal anwesend, alle mit persönlicher Schutzausrüstung
  - Verwendung von Einmal-Produkten
- Keine Sputum-Induktion
- Keine manuelle Hyperinflation, wenn eine maschinelle Möglichkeit besteht

Bei physiotherapeutischen Interventionen betreffend Mobilisation, Training und Rehabilitation gelten im Wesentlichen dieselben Sicherheitsvorkehrungen. Therapeutisches Equipment muss nach dem Gebrauch desinfiziert oder entsorgt werden, und die persönlichen Schutzmaßnahmen müssen nach lokaler Vorgabe eingehalten werden.

Diese kurze Zusammenfassung der wichtigsten Maßnahmen basiert auf einem aktuellen Artikel [109].

### Pneumologische Pflege

Die Versorgung von COVID-19-Patienten oder Personen mit COVID-19-Verdacht stellt eine große Herausforderung für die Pflege dar. Der Bedarf an professioneller Pflege und Betreuung variiert je nach Krankheitsverlauf und Einschränkungen durch vorbestehende Komorbiditäten.

Für Pflegepersonen ist bei potenziell übertragbaren und bedrohlichen Infektionen die frühzeitige Erkennung erkrankter Personen und die strikte Einhaltung von Schutzmaßnahmen bei der Durchführung der Pflegehandlungen von immanenter Bedeutung. Diese Schutzmaßnahmen dienen dem Selbstschutz des Gesundheitspersonals, aber auch der Vermeidung nosokomialer Infektionen.

Die Schutzmaßnahmen umfassen [110–112]:

- Konsequente Einhaltung persönlicher Hygienemaßnahmen:
  - Häufiges Händewaschen
  - Vermeidung des Kontakts mit Augen, Nase und Mund
  - Niesen und Husten möglichst in ein Taschentuch, welches anschließend sofort entsorgt wird
  - Mindestabstand zu anderen Personen 2 m
  - Händehygiene in allen Bereichen des Gesundheitswesens
- Organisatorische Schutzmaßnahmen:
  - Reduktion und Lenkung der Patientenströme zur Vermeidung der Übertragung von Patient zu Patient
  - Versorgung der betroffenen Patienten mit einem Mund-Nasen-Schutz, wenn der Gesundheitszustand der Patienten es zulässt
  - Räumliche Distanzierung von Verdachtspatienten von anderen Personen, idealerweise durch Unterbringung in einem Isolationszimmer mit Unterdruckschleuse und eigener Nasszelle
  - Reduktion des Patiententransports auf ein notwendiges Minimum inklusive Vorinformation des Transportpersonals
  - Reduktion sozialer Kontakte (Besucher im Krankenhaus) durch Steigerung telekommunikativer Möglichkeiten, Beschränkung der Anzahl der Besucher und der Dauer des jeweiligen Besuchs inklusive Einweisung der Besucher auf die Hygienemaßnahmen
  - Information, Schulung und Einweisung des betroffenen Personals zu Schutzmaßnahmen und Beobachtung des jeweils eigenen Gesundheitszustands
  - Reduktion des Ein- und Ausschleusens für die Behandlung und Pflege der betroffenen Patienten durch Kohortierung von Betroffenen unter gewissen Voraussetzungen bzw. Planung und Zusammenlegung von patientennahe durchzuführenden Maßnahmen
  - Desinfektion und Reinigung patientennaher und kontaminierter bzw. kontaminationsgefährdeter Flächen und verwendeter Medizinprodukte durch Desinfektionsmittel mit zumindest begrenzt viruzider Wirksamkeit
- Personalschutzmaßnahmen/persönliche Schutzausrüstung:
  - Die Auswahl der geeigneten persönlichen Schutzausrüstung richtet sich nach Art und Umfang der Tätigkeiten, die an Patienten durchgeführt werden.
  - Durch die aktuell weltweite Nachfrage nach Materialien zur persönlichen Schutzausrüstung soll auf einen ressourcenschonenden Umgang mit den zur Verfügung stehenden Produkten geachtet werden (Möglichkeiten der Wiederverwendung unter Einhaltung der jeweiligen Herstellerangaben und Sicherstellung der korrekten Zwischenlagerung).
  - Das An- und Ausziehen der persönlichen Schutzausrüstung soll konsequent geübt werden; bei COVID-19-Patienten soll nur geschultes Personal eingesetzt werden.
  - In vollständig isolierten Bereichen (etwa eine gesamte Station in einem Krankenhaus) sollte darauf geachtet werden, dass Schichten von Pflegepersonen, die die Schutzausrüstung dauerhaft tragen, nicht länger als 3 bis maximal 4 h dauern, bevor eine Pause eingelegt werden kann; dies soll verhindern, dass sich druckbedingte Verletzungen durch die Schutzausrüstung bilden. Hydrokolloide zum Schutz der Haut haben sich hierbei ebenso bewährt.
  - Händedesinfektion mit Desinfektionsmitteln mit zumindest begrenzt viruzider Wirksamkeit vor Anlegen der Schutzausrüstung bzw. nach Ausziehen der Handschuhe und vor Verlassen des Zimmers, und nach den sonst üblichen Regeln der Händehygiene
  - Bei Tätigkeiten, die mit Aerosolproduktion einhergehen, sollen Atemschutzmasken getragen werden, die zumindest 95 % aller Partikel mit einem Durchmesser von >0,3 µm abhalten. Dies entspricht der FFP2-Klasse der in Europa verwendeten Klassifikation der Atemschutzmasken.
  - Die minimale persönliche Schutzausrüstung bei der direkten Versorgung von COVID-19-Patienten oder bei Verdacht auf das Vorliegen einer SARS-CoV-2-Infektion besteht aus FFP2-Masken, Schutzbrille oder Gesichtsschild, einem langärmeligen wasserabweisenden Schutzkittel und Einweghandschuhen.

Die Maßnahmen zur Eindämmung der Infektionen werden von großen Teilen der Bevölkerung getragen. Dies und auch die Verlaufsmöglichkeiten von COVID-19 bewirken bei Betroffenen ein Angstgefühl, welches durch die Isolations- und Schutzmaßnahmen noch verstärkt werden kann. Die Reduktion der Besuche bzw. das Besuchsverbot in Krankenhäusern, Pflegeheimen und Altenwohnheimen sind förderliche Faktoren für Angst, Vereinsamung und soziale Isolation. Das Dilemma zwischen der Isolation als Schutzmaßnahme und den sozialen, psychischen und physischen Folgen durch die Isolation ist Gegenstand zahlreicher Untersuchungen und auch vom Deutschen Ethikrat als ethischer Kernkonflikt in der aktuellen Situation beschrieben worden. Hier ist die vor allem von Pflegepersonen vorgenommene Unterstützung in Aktivitäten des täglichen Lebens neben der Beziehungsarbeit eine wesentliche Maßnahme zur Vermeidung negativer Folgen bei Betroffenen [113].

Maßnahmen zur Bewältigung der Angst von betroffenen und isolierten Patienten bedienen sich vor allem Methoden der Kommunikation. Diese ist allerdings durch die Abstandsregeln, die räumliche Distanzierung und das Tragen der persönlichen Schutzausrüstung erschwert. Vor allem Palliativsituationen, der Sterbeprozess und die Unterstützung von Angehörigen, die einen Menschen verloren haben und Abschied nehmen wollen, sind Situationen mit besonderen Herausforderungen für das Gesundheitspersonal. Aber auch für Menschen mit Demenz, Delir oder anderen psychischen Erkrankungen sind Isolationssituationen schwierig anzunehmen. Es gibt Ansätze zur Stabilisierung der häuslichen Versorgung von an Demenz erkrankten Personen. Auch Handlungsempfehlungen rund um die Dokumentation der Sterbephase sind verfügbar [114–116].

### Pneumologische Funktionsdiagnostik

Während der kritischen Phase der Pandemie sollten Ambulanzen und Lungenfunktionslabore nach Möglichkeit die Zahl der Untersuchungen reduzieren, um zu große Patientenansammlungen (beispielsweise im Wartebereich) zu vermeiden. Darüber hinaus ist derzeit ein erhöhter Aufwand für Desinfektions- und Reinigungsarbeiten nach jeder Untersuchung notwendig. Somit sollte aktuell die Notwendigkeit von pneumologischen Spezialuntersuchungen (beispielsweise Lungenfunktionsdiagnostik, Spiroergometrie, Belastungs-Blutgase) in jedem Fall besonders streng hinterfragt werden. Vor jeder Untersuchung sollten Patienten standardisiert nach Infektionssymptomen befragt werden. Im Zweifel sollte die Diagnostik nicht durchgeführt bzw. verschoben werden.

Patienten ohne Infektionssymptome können weiterhin untersucht werden, wobei während der Pandemie das im Raum anwesende Personal bei der Untersuchung einen Mund-Nasenschutz (FFP2- oder FFP3-Maske) tragen sollte. Patienten nach überstandener SARS-CoV-2-Infektion (kein Anhalt für prolongierte Infektion, keine Symptomatik, PCR 2‑mal negativ) können ebenfalls untersucht werden. Im Lungenfunktionslabor sollte für den Bedarfsfall aber auch erweiterte Schutzausrüstung (Schutzbrille, Schutzmantel) vorrätig sein.
